# TB disability and multimorbidity at the onset of treatment in Kenya, Uganda, Zambia and Zimbabwe

**DOI:** 10.5588/ijtldopen.25.0171

**Published:** 2025-05-12

**Authors:** The Kenya, Uganda, Zambia and Zimbabwe TB Disability Study Group listed by surname alphabetically:, S.A. Adakun, F.M. Banda, A. Bloom, M. Bochnowicz, J. Chakaya, R. Chimzizi, J.P. Dongo, C. Duri, A.D. Harries, I. Kathure, F.N. Kavenga, A.M.V. Kumar, Y. Lin, H. Luzze, I. Mbithi, M. Mputu, A. Mubanga, D. Mudoola, D. Nair, M. Ngwenya, S. Ntambi, P. Owiti, A. Owuor, P. Thekkur, C. Timire, E. Tweyongyere, M. YaDiul, R. Zachariah

**Affiliations:** ^1^ Mulago National Referral Hospital, Kampala, Uganda;; ^2^ University Teaching Hospital, Ministry of Health, Lusaka, Zambia;; ^3^ Free Lance, Public Health Specialist, Washington D.C, USA;; ^4^ Department of Medicine, Therapeutics, Dermatology and Psychiatry, Kenyatta University, Nairobi, Kenya;; ^5^ Respiratory Society of Kenya, Nairobi, Kenya;; ^6^ Ministry of Health, Long-term Exceptional Technical Assistance Project, Genesis, Lusaka, Zambia;; ^7^ International Union Against Tuberculosis and Lung Disease-Uganda Office, Kampala, Uganda;; ^8^ Directorate of Health Services, Harare City Council, Harare, Zimbabwe;; ^9^ International Union Against Tuberculosis and Lung Disease, Paris, France;; ^10^ Department of Clinical Research, Faculty of Infectious and Tropical Diseases, London School of Hygiene & Tropical Medicine, London, UK;; ^11^ Division of National TB, Leprosy and Lung Disease Programme, Ministry of Health, Nairobi, Kenya;; ^12^ Ministry of Health and Child Care, AIDS and TB Department, Harare, Zimbabwe;; ^13^ The Union South-East Asia Office, New Delhi, India;; ^14^ Yenepoya Medical College, Yenepoya (Deemed to be University), University Road, Deralakatte, Mangalore, India;; ^15^ National Tuberculosis and Leprosy Programme, Ministry of Health, Kampala, Uganda;; ^16^ National Tuberculosis and Leprosy Programme, Ministry of Health, Lusaka, Zambia;; ^17^ Free Lance, Public Health Specialist, New Delhi, India;; ^18^ Kenyatta National Hospital, Nairobi, Kenya;; ^19^ UNICEF/UNDP/World Bank/WHO Special Programme for Research and Training in Tropical Diseases (TDR), World Health Organization, Geneva, Switzerland.

**Keywords:** tuberculosis, sub-Saharan Africa, real-time operational research, SORT IT, universal health coverage

## Abstract

**BACKGROUND:**

We evaluated the practicality of integrating assessments on the burden of multimorbidity (including disability) and the effectiveness of referral pathways at the start of TB treatment across Kenya, Uganda, Zambia and Zimbabwe.

**METHODS:**

A cohort study conducted within national TB programmes.

**RESULTS:**

Assessments were conducted in 1,683 (92%) of 1,822 patients, taking a median time of 29 minutes (interquartile range:20–37). Regarding comorbidities, 567 (34%) had HIV infection, 141 (8%) had high-blood pressure, 101 (6%) had a mental health disorder and 65 (4%) had diabetes. The three most common risk factors were undernutrition in 622 (37%), probable alcohol dependence in 311 (18%) and cigarette smoking in 275 (16%). Disability (inability to walk 400m in six minutes) was observed in 316 of 1,545 (20%) patients. Overall, 1,305 (78%) patients had at least one comorbidity, risk factor and/or disability. Successful referral ranged from 85–100% for most conditions, except for those with occupational silica exposure and disability, where access to pulmonary rehabilitation services was suboptimal.

**CONCLUSIONS:**

A significant proportion of TB patients experienced multimorbidity, including disability, highlighting the need for integrated, patient-centered care and decentralized point-of-care services, particularly for pulmonary rehabilitation. This multi-country study offers a promising pathway towards achieving that goal.

Although more than 85% of individuals successfully complete first-line TB treatment, a substantial proportion experience long term complications and disability that reduce their health-related quality of life.^1-4^ TB survivors also face a higher risk of all-cause mortality compared with the general population, often due to non-communicable diseases like cardiovascular disease.^5^ Due to this high burden of morbidity and mortality, international clinical standards for assessing, managing and rehabilitating post-TB disease were introduced in 2021.^6^ However, national TB programmes (NTPs) have largely overlooked this issue, although there are exceptions. Two operational research studies in China’s NTP demonstrated the feasibility of assessing patients who complete TB treatment for disability, comorbidities and risk factors and referring those in need of further care.^7,8^ Similarly, an operational research study in the NTPs of Kenya, Uganda, Zambia and Zimbabwe confirmed the feasibility of these assessments in adults at TB treatment completion,^9^ with 72% of patients having at least one comorbidity, risk factor or disability (defined as inability to walk 400m in the 6-minute walk test [6MWT]). While two-thirds of eligible patients were referred, 80% of those with disability required referral outside their original health facility for pulmonary rehabilitation.^9^ These findings highlight the need to strengthen patient-centred care for individuals with TB. Specifically, they suggest that NTPs and related health services should integrate early assessments and referrals for comorbidities, risk factors and disability - including pulmonary rehabilitation - at the start or during TB treatment. This approach aims to reduce morbidity, improve TB treatment outcomes and enhance long–term quality of life.^10^ This recommendation aligns with World Health Organization (WHO) policy on TB–associated disability, which advocates for integrated care, focused on the prevention and early identification of impairments and disability, through systematic screening with effective referral mechanisms.^11,12^

This study aimed to assess the practicality of integrating assessments on the burden of multimorbidity (including disability) and the effectiveness of referral pathways at the start of TB treatment across Kenya, Uganda, Zambia and Zimbabwe. Specific objectives were to evaluate: i) time taken for assessments and referrals; ii) the proportion of patients with comorbidities, risk factors and disability; and iii) the proportion of those identified who were referred and linked to care.

## METHODS

### Study design

This was a cohort study conducted within the routine framework of NTP services in four African countries.

### Study sites and setting

The study was conducted in the health facilities previously included in the first post-TB assessment study in Kenya, Uganda, Zambia and Zimbabwe.^9^ Health facilities were chosen based on logistic convenience including a sufficient annual case load of TB patients and their availability for long-term follow-up. Eligible facilities also required on-site equipment for blood glucose testing and blood pressure measurement.

### Study population and sample size

The study population included consecutive patients aged ≥18 years who started treatment for any form of TB at 26 selected health facilities between June and December 2024. The estimated sample size per country was 426 patients, based on a 23% prevalence of disability,^9^ with a 4% absolute precision and a 95% confidence level. To account for a 10% loss to follow-up, the sample size was adjusted to 475 patients per country.

### Training of health professionals using an adapted SORT IT

Study personnel were trained using an adapted SORT IT (Structured Operational Research Training Initiative) model focusing on real-time implementation research.^13,14^ In May 2024, representatives from each country – including the NTP manager (or delegate), the NTP Monitoring and Evaluation Officer and the in-country study coordinator – attended a one-week face-to-face training in Harare, Zimbabwe. The training covered key operational research principles, patient assessments and referrals, data collection and quality-control using an EpiCollect5 mobile-based application. Participants adapted a generic research proposal to their country needs and developed standard operating procedures (SOPs) and an implementation plan. Upon returning to their countries, the teams trained local implementation teams. Within each facility, a designated focal person(s) (doctor, nurse or clinical officer) was responsible for conducting assessments, referring patients, performing follow-ups and collecting data using a structured proforma. At the study’s conclusion, the same study group collaboratively wrote this manuscript and developed country-specific policy briefs for dissemination to relevant stakeholders.

### Assessments for comorbidities, risk factors and disability

Focal persons conducted assessments and data collection immediately after patients started TB treatment, following the methodology used in the previous study.^9^ Comorbidities assessed included HIV infection, diabetes mellitus (DM), high blood pressure and mental health disorders. Patients were asked about prior diagnosis of these conditions. HIV status was obtained from the TB register or determined through testing if HIV status was negative or unknown. All patients underwent a random blood glucose (RBG) with a follow-up fasting blood glucose (FBG) test, if needed. Hyperglycaemia was defined as a FBG ≥7.0mmol/L (≥126 mg/dL) or a RBG ≥11.1 mmol/L (≥200 mg/dL),^15^ with patients referred to a DM clinic for confirmation. High blood pressure was diagnosed in patients with a systolic blood pressure ≥140 mmHg and/or diastolic blood pressure ≥90 mmHg.^16^ Probable depression was assessed using the Patient Health Questionnaire (PHQ-2),^17^ with scores ≥3 prompting referral to a counsellor or a mental health specialist. TB risk factors included tobacco smoking (smoking at least once in the last one month), probable alcohol dependence (CAGE questionnaire score ≥2),^18^ occupational silica exposure and recreational drug use. Undernutrition was defined as a body mass index (BMI) <18.5 kg/m^2^, calculated from measuring height and weight. Disability was assessed using the 6MWT, as a proxy for cardio-pulmonary function (aerobic capacity and endurance).^19^ Patients were instructed to walk around a measured track for six minutes with disability defined as covering <400m in that time.^20^ Socio-economic status was assessed through questions on employment, wages and work absenteeism. The time taken to complete all assessments, including the 6MWT, was recorded in minutes.

### Onward referrals for eligible patients

Focal persons offered advice or referred patients with comorbidities, risk factors and/or disability to appropriate services within the same institution or another facility in the same catchment area. Referral criteria followed those used in the previous study (see Supplementary Data).^9^

### Data collection, analysis and statistics

Individual patient data were collected using a pre-designed proforma, entered into EpiCollect5 and cross-checked by focal persons at implementation sites. The country study coordinator supervised the process, addressing data quality reports generated bi-weekly by a team at the Centre for Operational Research, The Union. Data were analysed using STATA® (version 16.0 StataCorp LLC). Continuous data were summarised as means with standard deviations (SD) or medians with interquartile ranges (IQR), while categorical data were summarised as frequencies and proportions. Univariable and multivariable generalised linear (modified Poisson regression) models were used to identify patient characteristics associated with disability. All the variables were included in the multivariable model except those with variance inflation factor >5 (multicollinearity). Crude and adjusted prevalence ratios (aPR) with 95% confidence intervals (CI) were used as measures of association.

### Ethics

The research protocol received approval from each National TB Programme and the following ethics committees; the Union Ethics Advisory Group (EAG 01/24; 17/01/2024); the Kenya Medical Research Institute Scientific and Ethics Review Unit (KEMRI/RD/22; 20/05/2024); Mulago Research Ethics Committee (MHREC 2024-154; 21/06/2024); University of Zambia Biomedical Research Committee (5316-2024; 28/05/2024) and the Medical Research Council of Zimbabwe (MRCZ/A/3194; 19/06/2024). Informed consent was obtained from all study participants.

## RESULTS

### Demographic and clinical characteristics and feasibility of assessments

In total, 1,683 (92%) of 1,822 patients initiated on TB treatment underwent assessment. Their characteristics are summarized in Table 1. The mean age was 37 years (SD ± 12) and 72% were male. Almost all patients lived in urban areas, with most engaged in some form of employment. Among patients, 95% had pulmonary TB, 62% were bacteriologically-confirmed, 90% were newly diagnosed and 98% had drug-susceptible TB. The median time taken to complete assessments was 29 minutes (IQR 20–37), with a breakdown shown in Table 1. Almost three quarters of those employed or studying reported being absent from work or school for at least one day before TB diagnosis, the most common reasons being symptom severity and/or needing time to visit the health facility (Table 2). About 81% of those formally employed, 77% of those self-employed, 67% of daily wage labourers and 54% of students reported that they were absent from work/school. The median duration of absenteeism was 7 days, with a median wage loss of USD $35. More than half of those absent from work were family breadwinners.

**Table 1.** Demographics and clinical characteristics of TB patients aged ≥18 years at initiation of TB treatment in selected health facilities in four African countries, June–December, 2024.

Characteristics	n (%)
Total	1,683
*Country of recruitment*	
Kenya	501 (30)
Uganda	488 (29)
Zambia	477 (28)
Zimbabwe	217 (13)
*Age (in years)*	
18–29	494 (29)
30–44	782 (46)
45–59	330 (20)
≥60	77 (5)
*Sex*	
Male	1,204 (72)
Female	479 (28)
*Residence*	
Urban	1,646 (98)
Rural	37 (2)
*Occupation before onset of symptoms*	
Unemployed	348 (21)
Employed (Formal)	354 (21)
Self-employed (own business/farm)	509 (30)
Daily wage labourer (Informal)	347 (21)
Homemaker	51 (3)
Student	61 (4)
Retired	13 (1)
*Site of TB*	
Pulmonary	1,607 (95)
Extra pulmonary	76 (5)
*Type of TB*	
Bacteriologically confirmed	1,047 (62)
Clinically diagnosed	636 (38)
*Category of TB*	
New	1510 (90)
Previously treated	173 (10)
*Drug-susceptibility*	
Sensitive	1,657 (98)
RR/MDR-TB	26 (2)
*Duration for TB disability assessment (in minutes)^a^*	
<15	101 (6)
15–29	744 (44)
30–44	671 (40)
≥45	167 (10)

a Time taken to perform assessments of comorbidities, risk factors and disability

RR = rifampicin resistant; MDR = multi-drug resistant

**Table 2.** Work absenteeism, reasons, duration of absenteeism and wage loss before TB diagnosis among patients aged ≥18 years who were initiated on TB treatment and were assessed for comorbidities, risk factors and disability in selected health facilities in four African countries, June–December, 2024.

Characteristics	n (%)
Total^a^	1,271
*Absent from work/school*	
Yes	941 (74)
No	330 (26)
*Reason for being absent* (N=941)^b^	
Severity of symptoms	739 (79)
To visit health facility	512 (54)
Advised to take sick leave by doctor	61 (7)
Patient’s fear of spreading infection	27 (3)
Compulsory sick leave from workplace	11 (1)
Others	8 (1)
Median (IQR) days of absenteeism before TB diagnosis	7 (3–20)
Median (IQR) wage loss (in USD) due to absenteeism among those who were absent^c^ (N=908)	35 (14–114)
*Whether the patient who was absent from work was the primary bread winner of the family^c^* (N=908)	
Yes	508 (56)
No	400 (44)

a Includes those who were employed (354), self-employed (509), a daily wage labourer (347) and a student (61);

b Several patients had multiple reasons for being absent and numbers do not add up to 941.

c Multiple reasons are possible and total percentage does not add up to 100%; Includes those who are employed (354), self-employed (509) and a daily wage labourer (347).

### Symptoms, comorbidities, risk factors and disability

Prevalence of symptoms, comorbidities, risk factors and disability are shown in Table 3. There was a high prevalence of comorbidities. Over one-third of patients had HIV infection. While fewer than 10% had either DM, high blood pressure or mental health disorder, 75% (229/307) of these three conditions were newly diagnosed at assessment. The prevalence of risk factors was similarly high, with undernutrition (37%), probable alcohol dependence (18%) and smoking (16%) being the most common. There were 138 (8%) patients unable to perform the 6MWT due to weakness (56%), lower limb ailments (17%), being inpatient or bedridden (10%), breathlessness (9%) and other reasons (8%). About 18% of those aged >60 years were unable to perform 6MWT. Of the 1545 (92%) patients who performed the 6MWT, 316 (20%) walked <400m in 6 minutes. Overall, 1305 (78%) patients had at least one comorbidity, risk factor and/or disability.

**Table 3.** Symptoms, comorbidities, risk factors and disability in TB patients aged ≥18 years at initiation on TB treatment in selected health facilities in Kenya, Uganda, Zambia and Zimbabwe, June–December, 2024.

Category	Variable	n (%)
Total		1,683
*TB symptoms*	None	85 (5)
	Any symptom^a^	1,598 (95)
	Cough	1,453 (86)
	Fever	841 (50)
	Significant weight loss	1,022 (61)
	Night sweats	937 (56)
	Shortness of breath	712 (42)
	Tiredness / fatigue	863 (51)
	Chest pain	1,034 (61)
	Other^b^	191 (11)
*Comorbidities*		
HIV status	Positive	567 (34)
	Negative	1,116 (66)
*Diabetes Mellitus (DM)/ hyperglycemia*	Already known	28 (2)
	Tested for DM with either RBG/FBG	1,626 (97)
	Newly detected hyperglycemia	37 (2)^c^
	Prevalent DM/ hyperglycemia (already known and new)	65 (4)
*High blood pressure*	Already known	37 (2)
	Newly detected	104 (6)^d^
	Prevalent high blood pressure (already known and new)	141 (8)
*Mental health disorder*	Already known	13 (1)
	Newly detected with probable depression	88 (5)^d^
	Prevalent mental health disorder (already known and new probable depression)	101 (6)
*Risk factors*		
Probable alcohol dependence	CAGE score ≥2	311 (18)
Undernutrition	BMI<18.5 kg/m^2^	622 (37)
Silica dust	Occupational exposure	72 (4)
Smoked tobacco	Anytime in last one month	275 (16)
Recreational drug use	Current use^e^	106 (6)
Disability		
6-minute walk test (6MWT)	Done^f^	1,545 (92)
	6MWT <400 meters	316 (20)
*Multimorbidity*		
Comorbidity and/or risk determinant and/or disability	None	378 (22)
	One	543 (33)
	Two	422 (25)
	Three and above	340 (20)

a Some patients had multiple symptoms.

b Other symptoms such as myalgia, joint pain, abdomen discomfort and numbness of hands/feet.

c Percentages calculated with those not already known to have diabetes as denominator.

d Percentages calculated with those who were not already known to have the condition and assessed for the condition as denominator.

e Recreational drugs included marijuana, trihexyphenidyl, kuber (chewable nicotine).

f 1,545 patients underwent 6MWT among 1,683 patients recruited.

RBG = random blood glucose; FBG = fasting blood glucose; CAGE = cut, annoyed, guilty, eye-opener; BMI = body mass index.

### Characteristics associated with disability

Characteristics associated with disability (6MWT <400m) are shown in Table 4. On adjusted analysis, the significant associations were female sex (aPR 1.4, 95%CI 1.1–1.7), drug-resistant TB (aPR 1.7, 95%CI 1.0–2.9), HIV infection (aPR 1.3, 95%CI 1.1–1.6), mental health disorder (aPR 1.7, 95%CI 1.3–2.3), undernutrition (aPR 1.8, 95%CI 1.5–2.1) and occupational silica exposure (aPR 1.7, 95%CI 1.3–2.4). A linear trend in disability prevalence was observed with an increasing number of multimorbidities (univariable analysis).

**Table 4.** Characteristics associated with disability (6MWT <400 metres) in TB patients aged ≥18 years initiated on TB treatment in selected health facilities in Kenya, Uganda, Zambia and Zimbabwe, June–December, 2024.

Variable	Total	6MWT <400m	Crude	Adjusted*
	N	n (%)	PR (95% CI)	aPR (95% CI)
Total	1545	316 (20)		
*Age in years*				
18-29	464	90 (19)	1	1
30-44	719	134 (19)	1 (0.8-1.2)	0.9 (0.7-1.2)
45-59	299	74 (25)	1.3 (1.0-1.7)	1.2 (0.9-1.6)
≥60	63	18 (29)	1.5 (1.0-2.3)	1.6 (1.0-2.5)
*Sex*				
Male	1124	216 (19)	1	1
Female	421	100 (24)	1.2 (1.0-1.5)	1.4 (1.1-1.7)
*Site of TB*				
Pulmonary	1475	296 (20)	0.7 (0.5-1.1)	0.7 (0.4-1.1)
Extrapulmonary	70	20 (29)	1 (1.0-2.1)	1
Type of TB				
Bacteriologically confirmed	951	204 (21)	1	1
Clinically diagnosed	594	112 (19)	0.9 (0.7-1.1)	1.2 (0.7-2.1)
*Category of TB*				
New	1384	277 (20)	1	1
Previously treated	161	39 (24)	1.2 (0.9-1.6)	0.9 (0.7-1.1)
*Drug-susceptibility*				
Sensitive	1521	306 (20)	1	1
Resistant	24	10 (42)	2.1 (1.3-3.4)	1.7 (1.0-2.9)
*HIV status*				
Positive	505	124 (25)	1.3 (1.1-1.6)	1.3 (1.1-1.6)
Negative	1040	192 (18)	1	1
*Diabetes Mellitus/ hyperglycemia*				
Yes	56	15 (27)	1.3 (0.8-2.1)	1.3 (0.9-2.1)
No	1489	301 (20)	1	1
*High blood pressure*				
Yes	130	23 (18)	0.9 (0.6-1.3)	0.8 (0.5-1.2)
No	1415	293 (21)	1	1
*Mental health disorder*				
Yes	92	34 (37)	1.9 (1.4-2.5)	1.7 (1.3-2.3)
No	1453	282 (19)	1	1
*Probable alcohol Dependence*				
Yes	288	71 (25)	1	1
No	1257	245 (19)	1.3 (1.0-1.6)	1.1 (0.8-1.4)
*Undernutrition*				
Yes	554	155 (28)	1.7 (1.4-2.1)	1.8 (1.5-2.1)
No	991	161 (16)	1	1
*Occupational exposure to silica*				
Yes	71	30 (42)	2.2 (1.6-2.9)	1.7 (1.3-2.4)
No	1474	286 (19)	1	1
*Smoked tobacco*				
Yes	263	70 (27)	1.4 (1.1-1.7)	1.3 (1.0-1.8)
No	1282	246 (19)	1	1
*Recreational drug use*				
Yes	101	24 (24)	1.2 (0.8-1.7)	1.0 (0.6-1.5)
No	1444	292 (20)	1	1
*Multimorbidity (excluding 6MWT)*				
None	397	42 (11)	1	
One	556	109 (20)	1.9 (1.3-2.6)	
Two	362	90 (25)	2.4 (1.7-3.3)	
Three and above	230	75 (33)	3.1 (2.2-4.3)	

* Included all the variables except multimorbidity due to multicollinearity (variance inflation factor=23); the aPR figures in bold are statistically significant (p value <0.05).

PR = Prevalence ratio; aPR = Adjusted prevalence ratio; 6MWT = Six Minute Walk Test; CI = Confidence Interval.

### Referral for care

Eligibility and referral outcomes are shown in Table 5. Between 88–100% of those eligible for referral were referred to care. For most conditions, 88–100% were referred within the same facility, with the majority initiating care on the same day. Notable exceptions were: a) patients with occupational silica exposure (42% referred externally) and b) patients with disability (51% referred externally). Of those referred externally, only 70% with each condition reached the referral facility, and of these, 84% (silica exposure) and 86% (disability) initiated care.

**Table 5.** Status of referral for further care in those identified with comorbidities, risk factors and disability among TB patients aged ≥18 years initiated on TB treatment in selected health facilities in Kenya, Uganda, Zambia and Zimbabwe, June–December, 2024.

Conditions	Eligible for referral^a^	Referred to care	Referred within the same facility	Arrived at the referred facility	Initiated on care	Duration in days from referral to treatment initiation	
	N	n (%)	n (%)^b^	n (%)^b^	n (%)^c^	Median	(IQR)^d^
HIV	128	128 (100)	128 (100)	122 (95)	122 (100)	13	(1–14)
Diabetes mellitus/hyperglycemia	55	55 (100)	47 (85)	51 (93)	47 (92)	0	(0–5)
Hypertension	127	123 (97)	118 (96)	117 (95)	112 (96)	0	(0–5)
Mental health disorder	94	91 (97)	86 (95)	81 (89)	77 (95)	0	(0–8)
Risk factors							
Probable alcohol dependence	311	296 (95)	283 (96)	274 (93)	266 (97)	0	(0–7)
Undernutrition	622	613 (99)	607 (99)	595 (97)	571 (96)	0	(0–1)
Occupational exposure to silica	72	64 (89)	37 (58)	45 (70)	38 (84)	2	(0–8)
Smoking	275	257 (93)	244 (95)	234 (91)	221 (94)	0	(0–7)
Recreational drug use	106	99 (93)	93 (94)	88 (89)	83 (94)	0	(0–7)
Disability							
6MWT <400 metres	316	277 (88)	135 (49)	193 (70)	166 (86)	2	(0–8)

a For diabetes mellitus, hypertension and mental health disorder, patients who were newly diagnosed and patients who were known to have the condition but not on care or had uncontrolled disease were considered eligible for referral;

b Percentages calculated with total referred to care which includes within the same facility or outside the same facility for the condition as denominator;

c Percentages calculated with total arriving at the referral facility as denominator;

d Zero refers to initiation of treatment on the same day of the assessment

6MWT = six minute walk test.

## DISCUSSION

This is the first implementation research study to assess the feasibility and added value of incorporating evaluations of multimorbidity burden (including disability) and the effectiveness of referral pathways at the initiation of TB treatment within the NTP of four African countries. There were four key findings.

First, health care workers successfully integrated assessments into routine care, by assessing >90% of patients consecutively initiating TB treatment with a median assessment time of 29 minutes – comparable to post-TB assessment durations reported in China and Africa.^7,9^

Second, these findings are notable. Over three-quarters of patients had multimorbidity, which, with timely referral and care, can be managed. Notably most people with DM, high blood pressure and mental health disorders were newly diagnosed through these assessments. This is critical because TB patients with DM face higher mortality risks and an increased likelihood of recurrent TB, which is more often multidrug-resistant.^21,22^ Good glycaemic control, particularly with metformin use can reduce mortality.^23^ Similarly, hypertension in sub-Saharan Africa is associated with higher mortality both during and after TB treatment.^24^ There was a high prevalence of undernutrition, probable alcohol dependence and cigarette smoking, all of which negatively impact TB outcomes. Moderate to severe undernutrition is a risk factor for early TB-related death,^25^ although randomised controlled trials on the benefits of nutritional interventions remain limited. However, it is logical that national TB programs prioritize nutrition support for those identified with undernutrition. Smoking and excessive alcohol consumption during treatment increase the risk of death and treatment failure^26,27^ and several studies have shown that targeted interventions (for example, smoking cessation) are feasible and can improve treatment outcomes.^28^ Additionally, one-in-five of our TB patients was identified as disabled, although the true prevalence is likely higher as nearly 10% could not perform the 6MWT. Several risk factors were identified for disability, and this information can help facilities with a high TB burden to prioritize patients for disability assessment through the 6MWT.

Third, most patients identified with multimorbidity were successfully referred for further care, typically within the same health facility. In our previous study, intra-facility referrals in these same health facilities were sub-optimal for probable depression and behavioural risk factors, partly due to underutilisation of on-site HIV counsellors. ^9^ However, this has improved with HIV counsellors now engaged to counsel and manage probable depression and behavioural risk factors. There were two key exceptions to intra-facility referral. A substantial proportion of those with occupational silica exposure and disability required external referrals, which led to lower referral completion and care initiation. These patients need pulmonary rehabilitation, which has been shown to improve quality of life, exercise tolerance and lung function.^6,29,30^ However, such services are generally lacking at the primary healthcare level. Addressing this gap requires scaling up simplified, point-of-care community-based rehabilitation programs.^29,30^

Finally, it was disheartening to note that a high numbers of patients, particularly the self-employed and daily wage labourers, had to stop working and lose income even before being diagnosed with TB. More than half were family breadwinners. Absenteeism was less common amongst the self-employed and daily wage labourers, possibly because their financial needs were more pressing than those in formal employment. However, continuing to work while infected with TB can delay diagnosis and increase transmission within communities.

There were several strengths to this study. It is operationally relevant and responds to an identified research priority, both at the national and international level.^11^ The study also followed the Strengthening The Reporting of Observational Studies in Epidemiology (STROBE) guidelines.^31^ However, there were some limitations. In Zimbabwe, we did not reach the target sample size, mainly because of lower patient numbers in health facilities. Nearly 10% of our patients did not perform the 6MWT often due to weakness, breathlessness and being bedridden. As such, we have underestimated disability as judged by the 6MWT. Additionally, we lacked data on the type, length or quality of care received after referrals, as well as patient outcomes. Despite these limitations, our findings have an important overriding policy and practice implication. We have demonstrated that conducting multimorbidity assessments at the start of TB treatment is both feasible and valuable. Our findings are consistent with a previous systematic review and meta-analysis highlighting the high prevalence of TB related disability.^32^ To improve TB treatment outcomes, reduce the risk of recurrent TB, and enhance long-term survival, it is critical to integrate holistic, person-centred care that effectively identifies and manages coexisting conditions including disability.^29,35^ Encouragingly, this implementation research demonstrates that this can be done within existing healthcare services.

In conclusion, across four African countries a substantial proportion of patients initiated on TB treatment were found to have comorbidities, risk factors and disability. These findings reinforce the need for better integrated, patient-centred care for individuals with TB. Our study provides a promising pathway towards achieving this goal.

## Supplementary Material


