# Supplementary material for: TB disability and multimorbidity at the onset of treatment in Kenya, Uganda, Zambia and Zimbabwe
Source: IJTLD Open. 2025 May 12;2(5):291–8. doi: 10.5588/ijtldopen.25.0171 (PMC12068455; doi:10.5588/ijtldopen.25.0171)
Supplement: Supplementary file 1 [file ijtldopen25-0171_supplementarydata1.pdf]

Supplementary Annex: Referral criteria for further care in TB patients aged ≥18 years who started TB treatment and were identified with comorbidities, risk determinants and disability in selected health facilities in Kenya, Uganda, Zambia and Zimbabwe, June to December 2024.

Condition	Referral criteria
**Comorbidities**	
HIV infection	• Known and newly diagnosed people living with HIV not on anti-retroviral treatment
Diabetes mellitus / hyperglycemia	• Known diabetes mellitus not in care<br>• FBG ≥ 7 mmol/l in a new person or in DM care **OR** RBG ≥11 mmol/l in a new person or in DM care
High blood pressure	• Known high blood pressure not in care<br>• SBP ≥140mmHg **OR** DBP ≥90mmHg in a new person or in care for high blood pressure
Mental health disorder	• Known mental health disorder not in care<br>• PHQ-2 score ≥3 in a new person or in mental health care
**Risk factors**	
Probable alcohol dependence	CAGE score ≥2
Malnutrition	BMI <18.5 kg/m^2
Occupational exposure to silica	Any occupational exposure to silica
Smoking	Any tobacco smoking in last one month
Recreational drug use	Current recreational drug or substance use
**Disability**	
6MWT	Walked less than 400 metres

Abbreviations: TB- Tuberculosis; HIV- Human Immunodeficiency Virus; RBG- Random Blood Glucose; FBG- Fasting Blood Glucose; CAGE- Cut, Annoyed, Guilty, Eye-opener; BMI- Body Mass Index; SBP- Systolic Blood Pressure; DBP- Diastolic Blood Pressure; PHQ- Patient Health Questionnaire
